# Characteristics of TPT initiation and completion among people living with HIV

**DOI:** 10.5588/ijtldopen.23.0194

**Published:** 2024-01

**Authors:** L. Gunde, A. Wang, D. Payne, S. O’Connor, A. Kabaghe, N. Kalata, A. Maida, D. Kayira, V. Buie, L. Tauzi, A. Sankhani, A. Thawani, E. Rambiki, A. Ahimbisibwe, T. Maphosa, K. Kudiabor, R. Nyirenda, J. Mpunga, K. Mbendera, P. Nyasulu, F. Kayigamba, M. Farahani, A. C. Voetsch, K. Brown, A. Jahn, B. Girma, K. Mirkovic, MPHIA Survey Team

**Affiliations:** 1 U.S. Centers for Disease Control and Prevention (CDC), Lilongwe, Malawi; 2 U.S. CDC, Atlanta, USA; 3 Lighthouse Trust, Lilongwe, Malawi; 4 Elizabeth Glaser Pediatric AIDS Foundation (EGPAF), Lilongwe, Malawi; 5 Department of HIV/AIDS, STI and Viral Hepatitis, Ministry of Health, Lilongwe, Malawi; 6 National TB and Leprosy Elimination Program, Ministry of Health, Lilongwe, Malawi; 7 International Training & Education Center for Health (I-TECH) Department of Global Health, University of Washington, Lilongwe, Malawi; 8 ICAP Global Health, Lilongwe, Malawi; 9 ICAP at Columbia University, New York, USA

**Keywords:** tuberculosis, epidemiology, prevention

## Abstract

**BACKGROUND:**

TB preventive treatment (TPT) reduces morbidity and mortality among people living with HIV (PLHIV). Despite the successful scale-up of TPT in Malawi, monitoring and evaluation have been suboptimal. We utilized the Malawi Population-Based HIV Impact Assessment (MPHIA) 2020–2021 survey data to estimate TPT uptake and completion among self-reported HIV-positive persons.

**METHODS:**

We estimated the proportion of HIV-positive respondents who had ever undergone TPT, and determined the percentage of those currently on TPT who had completed more than 6 months of treatment. Bivariate and multivariable logistic regression were performed to calculate the odds ratios for factors associated with ever-taking TPT. All variables were self-reported, and the analysis was weighted and accounted for in the survey design.

**RESULTS:**

Of the HIV+ respondents, 38.8% (95% CI 36.4–41.3) had ever taken TPT. The adjusted odds of ever taking TPT were 8.0 and 5.2 times as high in the Central and Southern regions, respectively, compared to the Northern region; 1.9 times higher among those in the highest wealth quintile, and 2.1 times higher for those on antiretroviral therapy >10 years. Of those currently taking TPT, 56.2% completed >6 months of TPT.

**CONCLUSION:**

These results suggest low TPT uptake and >6 months’ completion rates among self-reported HIV+ persons. Initiatives to create demand and strengthen adherence would improve TPT uptake.

## RÉSUMÉ

**CONTEXTE:**

Le traitement préventif de la TB (TPT) réduit la morbidité et la mortalité chez les personnes vivant avec le VIH (PVVIH). Malgré l’extension réussie du TPT au Malawi, le suivi et l’évaluation n’ont pas été optimaux. Nous avons utilisé les données de l’enquête MPHIA (*Malawi Population-Based HIV Impact Assessment*) 2020–2021 pour estimer l’adoption et l’achèvement du TPT parmi les personnes se déclarant séropositives.

**MÉTHODES:**

Nous avons estimé la proportion de répondants séropositifs qui avaient déjà subi un TPT et déterminé le pourcentage de ceux qui sont actuellement sous TPT et qui ont terminé plus de 6 mois de traitement. Une régression logistique bivariée et multivariable a été effectuée pour calculer les rapports de cotes des facteurs associés au fait d’avoir déjà pris un TPT. Toutes les variables étaient autodéclarées et l’analyse a été pondérée et prise en compte dans la conception de l’enquête.

**RÉSULTATS:**

Parmi les répondants séropositifs, 38,8% (IC 95% 36,4–41,3) avaient déjà pris du TPT. Les probabilités ajustées de prise de TPT étaient 8,0 et 5,2 fois plus élevées dans les régions du centre et du sud, respectivement, que dans la région du nord ; 1,9 fois plus élevées chez les personnes appartenant au quintile de richesse le plus élevé, et 2,1 fois plus élevées chez les personnes suivant une thérapie antirétrovirale depuis plus de 10 ans. Parmi ceux qui prennent actuellement un TPT, 56,2% ont terminé >6 mois de TPT.

**CONCLUSION:**

Ces résultats suggèrent un faible taux d’utilisation du TPT et des taux d’achèvement de >6 mois parmi les personnes déclarées séropositives. Des initiatives visant à créer une demande et à renforcer l’adhésion permettraient d’améliorer l’utilisation du TPT.

Approximately one quarter of the world’s population is estimated to be infected with TB, and approximately 5–10% of those infected develop active TB disease in their lifetime.^1^ People living with HIV (PLHIV) are at a higher risk of developing active TB, and of dying from TB.^2^ TB preventive treatment (TPT) has been shown to reduce both TB morbidity and overall mortality among PLHIV.^3,4^ Persons at high risk of acquiring and developing active TB, such as PLHIV, those in whom TB disease has been ruled out and those who do not have any other documented contraindications, are eligible for TPT.^1^ Malawi has seen decreasing TB incidence over the last decade; however TB-HIV coinfection percentage is still high at 45%, with an HIV-positive TB incidence rate of 60 per 100,000 population (range: 30−99).^5^

The WHO first recommended TPT for PLHIV on antiretroviral therapy (ART) in 2011, and later revised these guidelines in 2015, providing guidance for resource-constrained settings for the implementation of lifelong (at least 36 months) isoniazid preventive therapy (IPT) for PLHIV.^6^ Clinical standards were recently published to complement WHO guidance on the implementation of TPT.^7^ Additional non-isoniazid (H, INH) only TPT regimens have subsequently become available for PLHIV, primarily 3HR, i.e., 3 months of INH plus rifampin (R, RIF) and 1HP or 3HP, i.e., 1 or 3 months of INH plus rifapentine (P, RPT).^1,2^ Different TPT options may be recommended based on the availability of appropriate formulations, consideration by age and population, safety, drug–drug interactions, limited resource implications, as well as factors that may affect completion, such as pill burden, dosing interval, and regimen duration.^1^ TPT remains a critical intervention to achieve the global targets of the End TB Strategy, as reiterated by the United Nations High-Level Meeting on TB in 2018, along with Malawi Ministry of Health (MOH) guidance.^8–10^ The U.S. President’s Emergency Plan for AIDS Relief (PEPFAR) strongly recommends that programs prioritize TPT for all eligible PLHIV.^11^

TPT was first included in the Malawi integrated clinical HIV guidelines in 2011 for PLHIV not on ART.^12^ This pre-ART TPT program ended after the introduction of universal ART in 2016. In 2016, TPT was recommended for PLHIV on ART following the revised 2015 WHO IPT guidance. Since September 2017, the Malawi MOH scaled up lifelong IPT for all ART patients in five of 28 districts with the highest TB-HIV burden: Lilongwe, Blantyre, Thyolo, Zomba, and Chiradzulu, representing the Central and Southern Regions. Implementation was supported by the Global Fund, PEPFAR, and PEPFAR-implementing partners.^13^ Between late 2019 and mid-2020, the TPT policy transitioned from continuous lifelong IPT in high-burden districts to 6-months’ IPT (6H) in all districts, and 3HP was introduced only for patients newly starting ART.^14^ TPT is currently offered to new-to-care PLHIV, within a package of preventive services, as well as TB contacts under 5 years regardless of HIV status. For new-to-care PLHIV, both TPT and ART adherence and side effects are monitored monthly for the first 3 months. However, ART appointments are aligned with TPT dispensing and clinical review visits within differentiated service delivery models.

Despite a successful TPT rollout in Malawi, there are gaps in TPT monitoring and evaluation systems. According to MOH program reports, facility-level TPT coverage among ART patients was determined from their TPT prescription at the most recent clinic visit. At facilities with electronic medical record system, this was measured from electronic dispensing records. At facilities with paper records, TPT dispensing was reviewed from patient treatment cards and reported as the proportion of patients retained on ART who received TPT at their most recent clinic visit. There were no program data on TPT dose adherence through pill counts or patient recall. Although TPT initiation and completion should be documented at PEPFAR-supported sites, data collection guidance has not been operationalized uniformly. The aim of this paper is to utilize Malawi Population-Based HIV Impact Assessment (MPHIA) 2020–2021 survey data to understand TPT uptake and completion among self-reported HIV-infected participants. The MPHIA was designed to estimate the proportion of persons who self-reported being HIV-infected, initiating and completing a 6-month course of TPT. We also analyzed factors associated with TPT uptake. Understanding characteristics of TPT uptake and completion is helpful to tailor TPT demand creation and adherence support interventions.

## METHODS

### Study design

MPHIA 2020–2021 was a nationally representative, cross-sectional survey with a two-stage cluster sampling design. Details of study design and sampling have been previously documented.^15^ Individuals ≥15 years of age who had slept in the house the previous night, who were able to provide verbal consent or assent and had a parent or guardian willing to provide verbal permission were all eligible for inclusion in the study.^15^

### Data collection

Participants were interviewed and demographic characteristics were collected, along with self-reported data on HIV status, initiation of TPT, and current use of TPT, along with months on TPT for those currently on TPT.

### Statistical analysis

We estimated the percentage of self-reported HIV-positive respondents who had initiated TPT, and among those currently on TPT, the percentage who completed at least 6 months of IPT. As respondents were not asked to specify the TPT regimen (i.e., 3HP or 6H), our definition of at least 6 months is only applicable to IPT and therefore a conservative estimate of completion rates. Data were weighted to account for differential selection probabilities, with adjustments for nonresponse and noncoverage of the population by age and sex. Weighted estimates are reported and 95% confidence intervals (CIs) were calculated using jackknife replicate weights. Results of χ^2^ tests comparing characteristics by TPT cohort with *P* < 0.05 were considered statistically significant. Bivariate and multivariable logistic regression were performed to calculate odds ratios (ORs) for factors associated with TPT initiation. STATA v16 (Stata Corp, College Station, TX, USA) was used for all analyses.

### Ethical review

All protocols were reviewed and approved by the Institutional Review Boards at the Centers for Disease Control and Prevention (Atlanta, GA, USA), Columbia University (New York, NY, USA), and the National Health Science Research Committee of Malawi (Lilongwe, Malawi).

## RESULTS

### Respondents’ characteristics

Of the eligible respondents (*n* = 30,049), 88.3% (*n* = 26,519) completed the MPHIA survey, among whom 7.5% (*n* = 2,320) self-reported HIV-infected and were asked if they had ever taken TPT (Figure). The majority who self-reported being HIV-infected were female (65.0%), under 44 years (59.5%), married or living together (64.3%), and living in the Southern Region (67.1%) (Table 1). High self-reported ART coverage was reported across all respondents (98.8%), with 99.3% of those who had ever taken TPT being currently on ART (Table 1). The median age of respondents who answered the TPT questions was 41 years (interquartile range [IQR] 34–49).

Most TPT users were female (64.5%), under 44 years (58.2%), married or living together (63.4%), from the Southern Region (68.9%) and on ART (99.3%). The distribution of demographic and clinical characteristics was similar among respondents who reported never taking TPT (Table 1). A slightly higher proportion of respondents who had ever taken TPT had attained secondary education or more than those who had never taken TPT (27.7% vs. 22.3%). The majority, 56.2% of those on TPT, were in the two highest wealth quintiles. A lower proportion of TPT users were currently pregnant than those who had never taken TPT (2.6% vs. 4.4%). TPT users were more likely to live in urban areas (30.3%) than those who had never taken TPT (23.3%). This was also reflected in higher percentages of those who had ever taken TPT living in urban cities – with 10.9% and 12.9% of those residing in Lilongwe City and Blantyre City, respectively.

### TPT initiation

Of those who were self-reported HIV-infected, 38.8% (95% CI 36.4–41.3) reported ever taking TPT (Figure, Table 2). Among those who had ever taken TPT, 29.1% (95% CI 25.4–33.2) self-reported currently taking TPT. Among those who self-reported being on ART, 39.5% (95% CI 37.0–41.9) reported initiating TPT, compared to 19.1% (95% CI 8.1–38.9) among those not currently on ART (Table 2). Among those who reported being on ART for ≥10 years, 45.8% (95% CI 40.9–50.8) reported initiating TPT. Almost half of those in the highest wealth quintile, 49.3%, reported ever taking TPT. Larger differences between ever taking TPT and currently taking TPT were found in the North than in the South (15.4%, 95% CI 10.9–21.4 vs. 39.9%, 95% CI 37.0–42.9; Table 2).

### Characteristics associated with TPT initiation

Region, wealth quintile, and years on ART were significantly associated with TPT initiation (Table 3). The adjusted odds of ever having initiated TPT were 7.96 and 5.24 times greater among those in the Central and Southern regions, respectively, compared to those in the North. The adjusted odds of TPT initiation among persons in the highest wealth quintile were 1.90 times that of those in the lowest wealth quintile in the adjusted model. Those who had been on ART for at least 10 years were 2.11 times more likely than those who had been on ART <1 year to have ever initiated TPT. Sex, age, marital status, urbanicity, travel time to healthcare facility, and breastfeeding status were not found to be associated with increased odds of TPT initiation.

### TPT completion

Among those who had ever taken TPT, 29.1% reported currently taking TPT at the time of the survey (Table 2).

Of those who reported currently taking TPT, 56.2% reported to have been on TPT for at least 6 months and were considered to have completed TPT (6H) for purposes of this analysis (Table 4). Those who had completed TPT tended to be older, more educated, married or living together, in urban areas, and not pregnant. The majority, 60.8%, of those who completed TPT resided in urban areas.

While there were no gender differences in TPT ever-use, there was a tendency for a higher TPT completion among females than in males (58.4%, 95% CI 48.3–67.9 vs. 52.4%, 95% CI 38.5–65.9). However, this difference was not statistically significant. Those who had been on ART for at least 1 year had a higher proportion of TPT completion.

## DISCUSSION

MPHIA survey results suggest that TPT uptake remains low among self-reported HIV-infected participants in Malawi. Only 38.8% of self-reported HIV-infected respondents reported having ever taken TPT. Because TB is the leading cause of death among PLHIV, low TPT uptake has implications for both ending TB and controlling the HIV epidemic.^1,2^ Malawi’s current national policy emphasizes the need to provide TPT to PLHIV who are newly starting ART, as TB incidence is highest in the first year of ART initiation and declines steeply in subsequent years.^16^ The survey results indicate that catch-up initiatives for existing patients would be needed to improve initiation and expand coverage among PLHIV.

Despite the recent shift to focus on PLHIV newly starting ART, TPT completion rates in this group have remained stagnant. The completion rates estimated in this analysis were comparable to those reported in routine program data, with slightly more than half of PLHIV on ART who initiate a course of TPT not completing it. These TPT completion rates are also similar to other studies on TPT completion and consistent with Malawi PEPFAR program data on TPT completion.^17,18^ This finding suggests the need for new initiatives including enhanced adherence support to improve TPT adherence and completion and to ensure ART clients attain the full level of protection from TB disease that TPT offers.

The observed relationship between TPT uptake and region is consistent with the scale up strategy in Malawi.^19,20^ All five of the districts initially prioritized for TPT rollout were in the Central and Southern regions. TPT expansion and demand creation efforts in the Northern region, such as 3HP prioritization, may be required to close the geographic gap in TPT uptake.

Understanding the association between time on ART and ever having initiated TPT is somewhat less intuitive. ART clients have more frequent clinical visits in the first 6 months of ART initiation.^20^ The observed association is most likely explained by the fact that the initial TPT scale up for ART patients started in 2017 in Malawi, primarily targeting all PLHIV on ART regardless of time on ART.^19^ This policy was changed between 2020 and 2021, targeting TPT to only PLHIV who are newly initiated on ART. We assume that clients who started ART in recent years were more likely to have initiated TPT. One possible explanation is that for an ART client who is eligible for TPT, every clinical encounter represents an opportunity to initiate TPT. This is similar to findings from Botswana where eligibility for TPT initiation is higher at visits post-ART initiation.^21^ Clients who have been on ART longer, therefore, had a greater cumulative chance of being initiated on TPT. There is also a possibility that clients who have been on ART longer would be more educated about HIV care, including about TPT and its benefits. Therefore, a client may have initially refused TPT but then decided to accept the treatment months or years later. Similarly, a client who was initially ineligible for TPT due to pregnancy or investigation for active TB disease may have deferred TPT initiation until a later clinical encounter. In such cases, TPT would be initiated when the client had been on ART for a longer period.

The observed association between TPT initiation and wealth is another example that highlights the complexity of characterizing factors associated with TPT uptake. While being in the highest income quintile was associated with uptake, this was not the case for urbanicity or level of education. Increased TPT uptake among the wealthiest respondents living with HIV could suggest that treatment cost was a barrier, but since TPT is available free of charge to all ART clients in the Malawi HIV program, other costs such as that for transport may be a barrier. A household study in Malawi previously found that the willingness to initiate TPT was also closely associated with household wealth.^22^ Further investigation is needed to better understand the driving forces behind this association, and how best to ensure that PLHIV of all income levels can access to TPT.

A limitation of our study is that the outcomes of interest, including HIV status, were self-reported. Respondents may not have accurately recalled whether they had ever taken TPT, particularly those who initiated long before the survey was conducted. Similarly, TPT completion estimates may have been affected by respondent recall when they started TPT. Only respondents who said they were currently on TPT were asked about time on TPT, which was used as a proxy measure, as TPT completion was not directly addressed in the survey. This precluded an analysis of completion among those who were not currently on TPT. Using 6 months on TPT as the cutoff for completion likely resulted in underestimated completion rates, as those on short-course TPT regimens would complete in less than 6 months.

The findings presented in this paper suggest low TPT uptake among self-reported HIV-infected participants in Malawi. This necessitates improved efforts to promote TPT demand creation, especially among those in the lower wealth quintiles, and offer TPT to all eligible PLHIV. Additionally, TPT in the Northern region should be prioritized to alleviate the geographic disparities in TPT uptake. Finally, given the reported low TPT completion rates, emphasis on TPT adherence and importance of course completion is required, including exploring integrating TPT and ART adherence support.

## Figures and Tables

**Figure.**
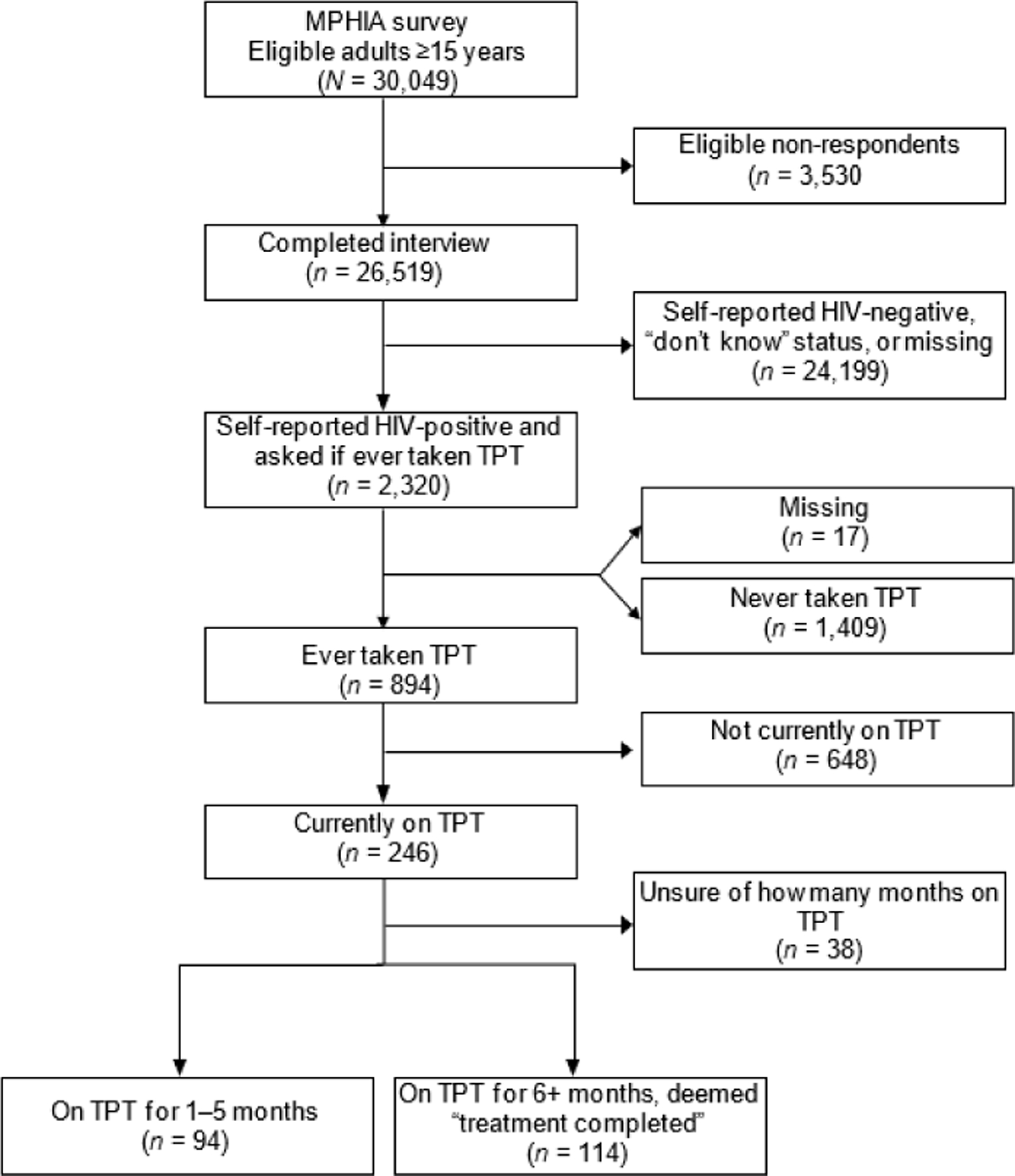
Flow diagram of MPHIA respondents in relation to taking TPT. MPHIA = Malawi Population-Based HIV Impact Assessment; TPT = TB preventive therapy.

**Table 1.** Demographic and clinical characteristics of people who self-reported HIV-positive (all) and those who were asked about TPT, Malawi Population-Based HIV Impact Assessment, 2020–2021.

	ALL self-reported HIV-positive (*n* = 2,303) %	Never taken TPT (*n* = 1,409) %	Ever taken TPT (*n* = 894) %
Demographic characteristics			
Sex			
Female	65.0	65.4	64.5
Male	35.0	34.6	35.5
Age group, years			
15–24	6.7	7.0	6.1
25–34	20.4	21.8	18.3
35–44	32.4	31.6	33.8
45–54	25.7	25.9	25.4
55–64	9.8	9.3	10.7
≥65	5.0	4.5	5.8
Education			
No education	13.2	13.5	13.0
Primary	64.4	67.5	59.5
Secondary or more	22.3	19.0	27.7
Marital status			
Never married	5.3	5.0	5.9
Married or living together	64.3	64.8	63.4
Divorced or separated	17.3	17.5	16.8
Widowed	13.1	12.6	13.9
Residence			
Urban	23.3	19.2	30.3
Rural	76.7	80.8	69.7
Regien			
North	8.7	12.0	3.5
Central	24.3	22.0	27.6
South	67.1	66.0	68.9
Zone			
North	8.7	12.0	3.4
Central-East	6.9	9.2	3.4
Central-West	10.7	9.1	13.3
Lilongwe City	6.6	3.9	10.9
South-East	26.4	30.4	20.1
South-West	32.6	30.4	35.9
Blantyre City	8.1	5.1	12.9
Wealth quintile			
Lowest	14.0	14.5	12.5
Second	15.6	17.3	12.9
Middle	20.6	21.9	18.4
Fourth	25.8	26.0	25.5
Highest	24.1	19.9	30.7
Clinical characteristics			
ART status			
On ART	98.8	98.4	99.3
Not currently on ART	1.2	1.6	0.7
Time on ART, years			
<1	10.6	12.2	8.2
1-<5	32.1	34.2	28.9
5-<10	30.4	29.7	31.5
≥10	26.9	24.0	31.4
Travel time (home to facility)			
<30 min	30.6	30.6	30.6
30–60 min	26.7	25.1	29.2
1–2 h	29.6	29.2	30.3
>2 h	13.1	15.2	9.9
Pregnancy/breastfeeding status			
Currently pregnant	4.4	5.1	2.6
Not pregnant	95.6	94.9	97.4
Currently breastfeeding	50.1	52.8	44.5
Not Currently breastfeeding	49.9	47.2	55.5

TPT = TB preventive therapy; ART = antiretroviral therapy.

**Table 2.** Demographic and clinical characteristics of self-reported HIV-positive respondents by TPT status, Malawi Population-Based HIV Impact Assessment, 2020–2021.

	Ever taken TPT		Currently taking TPT	
	*n*	Weighted % (95% CI)	*n*	Weighted % (95% CI)
Demographic characteristics				
Sex				
Female	1.608	38.5 (35.7–40.3)	617	29.4 (25.2–34.0)
Male	695	39.4 (35.6–43.3)	277	28.6 (21.8–36.4)
Age group, years				
15–24	143	35.5 (28.0–43 8)	53	24.6 (13.8–39.9)
25–34	445	34.7 (30.2–39.5)	153	36.1 (27.3–46 0)
35–44	788	40.4 (36.3–44 7)	318	27.4 (21.2–34.5)
45–54	581	38.1 (33.9–43.0)	228	25.8 (20.8–33.7)
55–64	226	42.1 (34.2–50.4)	92	29.7 (20.4–41.2)
≥65	114	45.0 (34.7–55.8)	50	31.3 (18.8–47.4)
Education				
No education	321	37.5 (31.7–43.8)	118	28.1 (20.3–37.5)
Primary	1,488	35.8 (32.9–38.9)	541	27.6 (23.3–32.3)
Secondary	445	47.4 (42.3–52.7)	210	33.5 (26.1–42 0)
More than Secondary	46	53.3 (38.5–67.5)	24	29.8 (13.5–53.6)
Marital status				
Never married	111	42.9 (34.3–51.9)	45	21.8 (10.5–40.0)
Married or living together	1,444	38.2 (35.4–41.2)	556	29.9 (25.2–35.1)
Divorced or separated	417	37.8 (32.5–43.5)	155	29.3 (21.7–38.3)
Widowed	331	41.1 (35.2–47.2)	138	28.4 (20.0–38.7)
Place of residence				
Urban	518	49.9 (43.7–56.2)	260	36.1 (29.6–43.2)
Rural	1,785	35.4 (32.7–38.2)	634	26.1 (21.8–31.0)
Regien				
North	164	15.4 (10.9–21.4)	25	38.6 (21.2–59.4)
Central	448	44.1 (39.0–49.3)	196	44.4 (36.0–53.1)
South	1,691	39.9 (37.0–42.9)	673	22.6 (18.8–26.9)
Zone				
North	164	15.4 (10.9–21.4)	25	38.6 (21.2–59.4)
Central-East	144	18.9 (12.4–27.8)	26	46.4 (22.2–72.4)
Central-West	155	48.2 (39.8–56.7)	74	48.4 (34.4–62.7)
Lilongwe City	149	63.7 (53.9–72.4)	96	38.8 (29.9–48.7)
South-East	678	29.5 (25.1–34.4)	203	18.6 (12.4–26.9)
South-West	821	42.8 (38.7–47.0)	353	21.3 (16.3–27.3)
Blantyre City	192	61.9 (55.6–67.8)	117	32.3 (23.7–42.3)
Wealth quintile				
Lowest	316	34.7 (29.1–40.7)	105	30.2 (21.5–40.7)
Second	358	32.1 (26.6–38.2)	115	25.6 (17.6–35.7)
Middle	488	34.5 (29.5–40.1)	173	21.7 (15.7–29.2)
Fourth	590	38.4 (33.6–43.4)	228	28.1 (21.0–36.4)
Highest	548	49.3 (44.3–54.4)	271	35.6 (29.2–42.6)
Clinical characteristics				
ART status				
On ART	2,249	39.5 (37.0–41.9)	885	29.4 (25.6–33.5)
Not currently on ART	28	19.1 (8.1–38.9)	6	0.0
Time on ART, years				
<1	208	30.4 (23.4–38.5)	64	49.3 (36.1–62.7)
1-<5	667	35.4 (31.3–39.7)	234	30.2 (23.8–37.4)
5-<10	657	40.6 (36.2–45.2)	262	28.9 (22.8–36.0)
≥10	574	45.8 (40.9–50.8)	266	26.0 (20.6–32.2)
Travel time (home to facility)				
<30 min	679	39.3 (34.8–44.0)	267	32.0 (25.3–39.5)
30–60 min	593	43.0 (38.4–47.7)	253	30.6 (24.0–38.1)
1–2 h	673	40.2 (35.8–44.7)	270	25.5 (19.0–32.1)
>2 h	294	29.6 (24.5–35.3)	88	28.4 (18.4–41.1)
Pregnancy/breastfeeding status				
Currently pregnant	71	25.0 (16.1–36.7)	18	39.1 (18.7–64.3)
Not pregnant	1,526	39.0 (36.2–41.9)	593	29.3 (25.0–33.9)
Currently breastfeeding	209	29.5 (23.2–36.7)	62	21.3 (12.7–33.4)
Not Currently breastfeeding	209	36.9 (30.4–43.8)	76	43.3 (31.6–55.7)
Total	2,303	38.8 (36.4–41.3)	894	29.1 (25.4–33.2)

TPT = TB preventive therapy; CI = confidence interval; ART = antiretroviral therapy.

**Table 3.** Characteristics associated with TPT initiation, Malawi Population-based HIV Impact Assessment, 2020–2021.

Characteristics	*n*	Ever taken TPT % (95% CI)	OR (95% CI)	*P*-value	aOR (95% CI)
Sex					
Female	1,503	38.1 (35.2–41.0)	0.96 (0.8–1.15)	0.661	
Male	632	39.5 (35.6–43.7)			
Age group, years					
15–24	141	36.4 (28.6–44.9)			
25–34	401	35.1 (30.1–40.3)	0.97 (0.65–1.44)	0.856	
35–44	727	39.3 (35.1–43.8)	1.23 (0.84–1.81)	0.276	
45–54	550	38.1 (33.4–43)	1.13(0.76–1.69)	0.541	
55–64	210	41.6 (33.2–50.5)	1.32 (0.83–2.11)	0.234	
≥65	106	47.6 (36.9–58.5)	1.48 (0.86–2.56)	0.148	
Education					
No education	294	38.7 (32.4–45.3)			
Primary	1,383	35.5 (32.4–38.6)	0.93 (0.7–1.23)	0.593	0.93 (0.66–1.31)
Secondary	423	47.4 (42–52.9)	1.5 (1.07–2.1)	0.019	1.29 (0.81–2.05)
More than Secondary	32	49.5 (33–66.2)	1.89 (0.98–3.65)	0.056	1.04 (0.38–2.85)
Marital status					
Never married	104	44.9 (35.8–54.4)			
Married or living together	1,327	38.2 (35.3–41.3)	0.83 (0.56–1.22)	0.318	
Divorced or separated	384	36.1 (30.7–42)	0.81 (0.54–1.23)	0.306	
Widowed	320	40.4 (34.4–46.8)	0.93 (0.59–1.47)	0.744	
Place of residence					
Urban	474	49.9 (43.6–56.1)			
Rural	1,661	35.1 (32.3–38.1)	0.55 (0.41–0.73)	<0.001	0.85 (0.52–1.41)
Regien					
North	153	15.4 (10.6–21.8)			
Central	411	44.1 (39–49.4)	4.32 (2.73–6.84)	<0.001	7.96 (4.31–14.71)
South	1,569	39.6 (36.5–42.7)	3.64 (2.37–5.58)	<0.001	5.24 (2.94–9.33)
Wealth quintile					
Lowest	292	35.6 (29.4–42.3)			
Second	335	32 (26.5–38.1)	0.89 (0.61–1.29)	0.526	1.11 (0.71–1.75)
Middle	453	33.1 (27.8–39)	1 (0.72–1.38)	0.982	1.11 (0.74–1.67)
Fourth	552	38.5 (33.5–43.7)	1.17 (0.84–1.64)	0.342	1.4 (0.94–2.09)
Highest	501	49.1 (44–54.2)	1.83 (1.32–2.54)	0.001	1.9 (1.12–3.24)
ART status					
Currently on ART	2,226	39.5 (37–42)	2.76 (0.98–7.76)	0.054	4.4 (0.63–30.98)
Not currently on ART	30	22.5 (11.7–38.8)			
Time on ART, years					
<1	184	32 (24.4–40.6)			
1-<5	620	34.6 (30.4–39)	1.25 (0.84–1.86)	0.253	1.26 (0.75–2.11)
5-<10	613	40.4 (35.9–45.2)	1.57 (1.04–2.37)	0.034	1.27 (0.76–2.13)
≥10	543	45 (40–50.2)	1.94 (1–28-2.93)	0.003	2.11 (1.23–3.62)
Travel time (home to facility)					
<30 min	679	39 (34.2–43.8)			
30 min-1 h	593	42.9 (38–47.8)	1.17 (0.89–1.52)	0.244	1.41 (0.99–2.01)
1–2 h	679	39.5 (34.8–44.1)	1.04 (0.79–1.36)	0.782	1.41 (1.01–1.98)
>2 h	294	30.5 (24.8–36.2)	0.65 (0.46–0.92)	0.017	0.92 (0.61–1.4)
Pregnancy Status					
Currently pregnant	63	23.1 (14.1–35.3)			
Not pregnant	1,434	38.5 (35.6–41.5)	1.91 (1.08–3.38)	0.027	1.45 (0.77–2.75)
Breastfeeding Status					
Currently breastfeeding	192	30.1 (23.4–37.7)			
Not currently breastfeeding	197	36.1 (29.5–43.4)	1.4 (0.93–2.09)	0.100	
Total	2,135	38.6 (36.1–41.2)			

TPT = TB preventive therapy; OR = odds ratio; CI = confidence interval; aOR = adjusted OR; ART = antiretroviral therapy.

**Table 4.** Time on TPT among self-reported HIV-positive respondents who reported being currently on TPT initiation by selected demographics and clinical characteristics, Malawi Population-Based HIV Impact Assessment, 2020–2021.

	*n*	1 =5 months % (95% CI)	≥6 months % (95% CI)	*P*-value
Sex				
Female	141	41.6 (32.1–51.7)	58.4 (48.3–67.9)	0.432
Male	67	47.6 (34.1–61.5)	52.4 (38.5–65.9)	
Age group, years				
15–24	11	62.9 (31.8–86.1)	37.1 (13.9–68.2)	0.141
25–34	43	54.5 (38.6–69.5)	45.5 (30.5–61.4)	
35–44	71	34.6 (22.0–49.7)	65.4 (50.3–78)	
45–54	48	41.3 (27.7–56.3)	58.7 (43.7–72.3)	
55–64	20	56.9 (32.3–78.5)	43.2 (21.5–67.7)	
≥65	15	31.3 (14.2–55.7)	68.7 (44.3–85.8)	
Educatioin				
No education	27	28.1 (13.5–49.6)	71.9 (50.4–86.6)	0.093
Primary	122	50.4 (39.9–61.0)	49.6 (39.0–60.2)	
Secondary	52	41.3 (27.6–56.5)	58.7 (43.5–72.4)	
More than secondary	7	14.1 (1.0–72.8)	85.9 (27.2–99.0)	
Marital status				
Never married	7	48.7 (11.1–87.9)	51.3 (12.1–88.9)	0.813
Married or living together	136	41.2 (30.5–52.7)	58.8 (47.3–69.5)	
Divorced or separated	32	49.1 (30.2–68.3)	50.9 (31.8–69.8)	
Widowed	33	48.9 (32.1–65.9)	51.1 (34.1–67.9)	
Place of residence				
Urban	81	39.2 (25.9–54.3)	60.8 (45.7–74.1)	0.414
Rural	127	46.7 (35.8–57.9)	53.3 (42.2–64.2)	
Region				
North	5	19.1 (3.0–63.9)	80.9 (36.1–97.0)	0.425
Central	73	40.7 (28.1–54.7)	59.3 (45.4–71.9)	
South	130	47.4 (35.5–59.5)	52.7 (40.5–64.5)	
Zone				
North	5	19.1 (3.0–63.9)	80.9 (36.1–97)	0.522
Central-East	10	51.3 (4.2–96.2)	48.7 (3.8–95.8)	
Central-West	29	33.3 (17.8–53.4)	66.7 (46.6–82.2)	
Lilongwe City	34	47.5 (34.1–61.4)	52.5 (38.7–65.9)	
South-East	27	48.7 (25.9–72.0)	51.3 (28.0–74.1)	
South-West	70	54.1 (39.3–69.2)	45.9 (30.8–61.8)	
Blantyre City	33	33.4 (16.0–56.9)	66.7 (43.1–84)	
Wealth quintile				
Lowest	24	39.9 (21.8–61.2)	60.1 (38.8–78.2)	0.218
Second	24	55.8 (32.8–76.6)	44.2 (23.4–67.2)	
Middle	30	37.6 (20.5–58.5)	62.4 (41.5–79.5)	
Fourth	46	56.1 (41.3–69.9)	43.9 (30.1–58.8)	
Highest	84	36 (23.1–51.3)	64 (48.7–77.0)	
ART status				
Currently on ART	208	43.8 (35.0–52.6)	56.2 (47.4–65.1)	
Not currently on ART				
Time on ART, years				
<1	28	69.4 (44.7–86.4)	30.6 (13.6–55.3)	0.067
1-<5	57	37.8 (24.4–53.4)	62.2 (46.6–75.6)	
5-<10	61	42.6 (27.9 58.7)	57.4 (41.4–72.1)	
≥10	51	37.2 (24.1–52.5)	62.8 (47.5–75.9)	
Travel time (home to facility)				
<30 min	69	51.4 (38.2–64.3)	48.6 (35.7–61.8)	0.506
30 min-1 h	60	44.2 (28.3–61.4)	55.8 (38.6–71.7)	
1–2 h	54	37.3 (24.9–51.5)	62.8 (48.5–75.1)	
>2h	22	37.5 (17.8–62.5)	62.5 (37.5–82.2)	
Pregnancy status				
Pregnant	6	67.9 (22.6–93.9)	32.1 (6.1–77.4)	0.176
Not pregnant	135	40.4 (31.1–50.5)	59.6 (49.6–68.9)	
Breastfeeding status				
Currently breastfeeding	25	50.3 (20.5–79.8)	49.7 (20.2–79.5)	0.986
Not Currently breastfeeding	10	50.6 (28.9–72.1)	49.4 (27.9–71.2)	
Total	208	43.8	56.2	

TPT = TB preventive therapy; CI = confidence interval; ART = antiretroviral therapy.
